# The hypermethylation of *p16* gene exon 1 and exon 2: potential biomarkers for colorectal cancer and are associated with cancer pathological staging

**DOI:** 10.1186/s12885-018-4921-5

**Published:** 2018-10-22

**Authors:** Xiaoxia Ye, Mingming Mo, Simin Xu, Qingjin Yang, Minhua Wu, Junjie Zhang, Bin Chen, Jian Li, Yu Zhong, Qionglin Huang, Chun Cai

**Affiliations:** 1 Department of Histology and Embryology, Guangdong Medical University, Zhanjiang, 524023 People’s Republic of China; 2 Analysis Center, Guangdong Medical University, Zhanjiang, 524023 People’s Republic of China; 3 Institute of Neurology, Affiliated Hospital, Guangdong Medical University, No. 2 Wenming Dong Road, Zhanjiang, Guangdong 524023 People’s Republic of China

**Keywords:** *p16* gene, Exon 1, Exon 2, DNA methylation, Colorectal cancer

## Abstract

**Background:**

Tumor suppressor gene *p16* promoter hypermethylation has been widely studied in colorectal cancer (CRC), yet its clinicopathological significance remains controversial. The methylation alterations of other regions within *p16* gene are still rarely researched. The present study aimed to explore the methylation changes of *p16* gene body in CRC and to find whether they were associated with clinicopathological staging of CRC.

**Methods:**

Paired colorectal cancer tissues and corresponding adjacent normal tissues from 30 CRC patients were collected. The methylation levels of two CpG islands within *p16* gene body, exon 1 and exon 2, were accurately assessed simultaneously by a LC-MS/MS method. The p16 protein expressions were assessed by immunohistochemistry assay. Statistical analyses were carried out using SPSS 17.0 software. Heat-map analysis was carried out by HemI 1.0 software.

**Results:**

In the present study, CRC tissues showed more highly methylated than adjacent normal tissues at both CpG islands of *p16* gene. And exon 2 hypermethylation was higher and more frequent than exon 1. The ROC curve analysis showed that the simultaneous use of both indicators had excellent sensitivity and specificity for distinguishing CRC tissues and adjacent normal tissues. Following, the methylation level of *p16* exon 1/2 was negatively related to p16 protein expression. Further correlation analysis revealed that *p16* exon 1 hypermethylation was associated with N/Dukes staging (*p* = 0.033), and *p16* exon 2 hypermethylaiton was associated with T staging (*p* = 0.035).

**Conclusions:**

The *p16* gene body was remarkably hyper-methylated in CRC tissues and associated with p16 protein expression and cancer clinicopathological staging. The combination of *p16* exon 1 and exon 2 could better reflect the overall methylation status of *p16* gene body and provide potential biomarkers of CRC.

## Background

Colorectal cancer (CRC) is the third most common malignant neoplasms in the world. Each year almost 1.4 million new cases were diagnosed and 0.7 million patients died of this disease [1]. Aberrant DNA methylation is an important driver mechanism in tumorigenesis [2] and ever-growing number of genes showed abnormal methylation in CRC [3–5]. Because aberrant methylation alteration can begin very early in tumor progression, especially earlier than protein expression changes and malignant cell proliferation [6], such genes are promising to be good indicators for early diagnosis and prognosis of CRC.

The *p16* gene (also named as *CDKN2A, INK4A, CDK4I*) is one of the most studied epigenetic markers in CRC. As a tumor suppress gene, *p16* inactivation results in loss of the cellular capacity to block cell cycle and has been widely reported in human malignancy [7, 8]. The *p16* hypermethylation is a frequent event in CRC and acts as a major mechanism leading to *p16* inactivation [7]. Since the methylation change of *p16* gene in cancer was firstly identified at promoter-associated region [9], previous most research of *p16* aberrant methylation focus on its promoter and/or upstream-exon1 regions. Concerning the change of *p16* promoter methylation in CRC, most investigators observed that the tumor tissues were more highly methylated than adjacent normal mucosae [10–15]. But a recent large-scale research found that nearly 10% of CRC cases had greater methylation at *p16* promoter region in the adjacent non-neoplastic tissues than in the carcinoma [16]. Regarding the impacts of *p16* promoter hypermethylation (PHM) on CRC, some investigations revealed a correlation between it and some clinicopathological parameters or poor prognosis [10–14, 16], such as *p16* PHM with larger tumor size, more frequent recurrence and shortened survival. But others did not observe statistically correlation [17–19]. Even some reported CRC patients with *p16* PHM had a better survival [20]. Due to those inconsistent results, the clinicopathological significance of *p16* PHM remains controversial. More optimal methylation loci within *p16* gene are still to be explored.

Recently, gene body methylation (GbM) was found that frequently occurred in the transcribed regions of many oncogenic regulated genes and actively involved in multiple regulation processes [21, 22]. More detailed genome-wide studies have demonstrated that GbM can alter gene expression by silencing alternative promoters or effecting transcription elongation or regulating splicing [23–25]. Thereby GbM is suggested as a novel biomarker or therapeutic target in cancer [26]. However, the intragenic DNA methylation of *p16* gene received less attention and is poorly understood up to date. A few studies explored the methylation status of *p16* exon 2 region and found it was frequently methylated in head and neck squamous carcinoma [27], oesophageal cancer [28] and breast cancer [29], and its methylation changes were associated with breast carcinogenesis. Whether hypermethylation of *p16* exon 2 also occurs in other cancers remains unclear.

To explore the methylation changes of *p16* gene body in CRC, we focused on CpG-rich regions in *p16* gene body, namely exon 1 and exon 2. Their methylation levels were evaluated in paired CRC and adjacent normal tissues by LC-MS/MS method, which can quantify the average methylation level of target genomic region [30]. Statistical analysis was carried out to find more reliable methylation biomarkers. Moreover, we analyzed the relationship between methylation status of each region and clinicopathological parameters of CRC patients, such as gender, age, differentiation and T/N/Dukes stage, to investigate whether they were associated.

## Methods

### Chemicals and reagents

Cytosine (Cyt), Adenine (Ade) and Protease K were purchased from Sigma (St. Louis, USA). Isotopes Cyt^13^C^15^N_2_ and Adenine-2-^13^C were purchased from Toronto Research Chemicals Inc. (Toronto, Canada) and C/D/N Isotopes Inc. (Quebec, Canada), respectively. PCR reagents were purchase from TAKARA Bio Inc. (Dalian, China). Ammonium formate, methanol, acetonitrile, formic acid (chromatographic grade) were purchased from Merck (Darmstadt, Germany). The monoclonal antibody against p16 protein and the Streptavidin-Peroxidase Detection Kit for immunohistochemistry were purchased from ZSGB Bio (Beijing, China).

### CRC tissue samples

Thirty pairs of colorectal cancer tissue and corresponding para-carcinoma tissue were collected from Department of gastrointestinal surgery, Affiliated Hospital of Guangdong Medical University from 2014 to 2015. The 30 patients comprised 18 males and 12 females, with a mean age of 56.5 years (range 20–77). The mean tumor size was 4.5 cm^3^. The adjacent tissues were about 10 cm distant from tumors. All CRC samples were confirmed by pathological diagnosis. Fresh tissues were snap frozen in liquid nitrogen and stored at − 80 °C until further protocols. Clinical data were collected prospectively. The collection of tissue samples for this project was approved by Ethic Censor Committee of Affiliated Hospital of Guangdong Medical University and manipulated fully in accordance with its guidelines.

### DNA extraction and bisulfite conversion

Genomic DNA was extracted from tissue samples using Tissue Genomic DNA Extraction Kit (Tiangen, Beijing, China) following the manufacture’s protocols. The concentration and purity of genomic DNA were determined using Nanodrop2000 Ultramicro Spectrophotometer (Thermo Scientific, Massachusetts, USA). 200 ng DNA was used for bisulfite conversion with accordance to the specification of EZ DNA Methylation-Gold Kit (ZYMO, Irvine, USA).

### PCR amplification and purification of target regions

The whole CpGs islands lying in the exon 1 and exon 2 within *p16* gene body were targeted and amplified from bisulfite-converted genomic DNA via nested PCR using specific modified primers (Table 1). The outer PCR amplification was conducted in a 25 μL total reaction volume containing 1.0 μL of 10 μM of each primer, 1 U *ExTaq* DNA polymerase, and approximately 75 ng bisulfite-treated genomic DNA. The inner PCR was performed in a 50 μL total reaction volume including 2.0 μL of 10 μM of each primer, 2 U *ExTaq* DNA polymerase and about 30 ng outer PCR products. PCR amplification was implemented in Veriti gradient thermal cycler (Applied Biosystem, Carlsbad, USA). The inner PCR products were evaluated using 1.5% agarose gel electrophoresis and bidirectionally sequenced with inner PCR primers to ensure the sequence correctness. Acquired PCR products of two target regions were purified according to the instruction of EZ gene Gel/PCR Extraction Kit (ZYMO, Irvine, USA), and their concentration was measured by Ultramicro Spectrophotometer. At the same time, three specific DNA samples with known methylation level (0%, 47% and 100%) were also prepared as controls according to our prior work [30].

**Table 1:** The primer sequences and PCR conditions

Primer name	Forward primers^a^ (5′-3′)	Reverse primers^a^(5′-3′)	Anneal temp.	Product
*p16* exon 1				
Outer	**TT**AGAGGATTTGAGGGA**T**AGGGT	T**A**CA**A**ACCCTCTACCCACCT**AA**AT	56 °C	324 bp
Inner	GGATTTGAGGGA**T**AGGGT	CCCTCTACCCACCT**AA**AT	56 °C	313 bp
*p16* exon 2				
Outer	TGG**T**AGGT**T**ATGATGATGGG**T**AG	**A**TCCTCACCT**A**A**AAA**ACCTTCC	54 °C	321 bp
Inner	GGT**T**ATGATGATGGG**T**AG	TTACT**A**CCTCT**AA**T**A**CCCC	53 °C	273 bp

^a^Sequence differences between modified primers according bisulfite-converted DNA and unconverted DNA are indicated in boldface type

### Methylation level determination of target regions by a LC-MS/MS method

The NQ-E (Nucleobases Quantitation of bisulfite amplicon coupled with an Equation) method described in a recent publication [30], was applied to determine the methylation levels of target regions. Briefly, 100 ng purified PCR products of *p16* exon 1/2 region were added into 100 μL of 100 ng/mL mixed internal standard solution including Cyt^13^C^15^N_2_ and Adenine-2-^13^C and mixed evenly, then dried at 60 °C. The residue was mixed with 200 μL of 88% formic acid (*v*/v) and hydrolyzed at 140 °C for 90 min. Hydrolyzed product was dried and dissolved in 200 μL acetonitrile - 0.7 mM aqueous ammonium formate (93, 7, v/v), and then centrifuged at 12,000 g for 5 min. The final supernatant was extracted for LC-MS/MS analysis.

After LC separation and MS detection, the quantification of cytosine (Q_Cyt-M_) and adenine (Q_Ade-M_) in target amplicons were accomplished in multiple reactions monitoring mode. Based on the LC-MS/MS data and Genebank data (P_Gua-D_ and P_Cyt-CpG-D_ in target genomic region), the average methylation level of target region was calculated using the following two formulas as described previously [30]:

1 $$ {\mathrm{P}}_{\mathrm{GuaCyt}\hbox{-} \mathrm{M}}={\mathrm{Q}}_{\mathrm{Cyt}\hbox{-} \mathrm{M}}/\left({\mathrm{Q}}_{\mathrm{Cyt}\hbox{-} \mathrm{M}}+{\mathrm{Q}}_{\mathrm{Ade}\hbox{-} \mathrm{M}}\right) $$

And

2 $$ \%\mathrm{Methylation}=\left({\mathrm{P}}_{\mathrm{Gua}\mathrm{Cyt}-\mathrm{M}}-{\mathrm{P}}_{\mathrm{Gua}-\mathrm{D}}\right)/{\mathrm{P}}_{\mathrm{Cyt}-\mathrm{CpG}-\mathrm{D}}\times 100\% $$

### Immunohistochemistry assay

Immunohistochemical analyses for p16 protein were performed in 30 CRC samples. The paraffin-embedded tissue sections were deparaffinized with xylene and rehydrated. For antigen retrieval, sections were immersed in 10 mM citrate buffer and microwaved for 5 min. Endogenous peroxidase and non-specific protein binding was blocked by incubating with 3% H_2_O_2_ and then with 10% goat serum. Then sections were incubated, in turn, with the anti-p16 primary antibody (dilution 1:200) at 4 °C overnight, with the biotin-labelled secondary antibody for 15 min and with HRP-labelled streptavidin for 15 min. Signals were visualized with DAB for 1 min, with slight counterstaining using hematoxylin. In each experiment, the primary antibody was omitted as negative controls. The sections were evaluated independently by two investigators as described previously with slight modification [31]. The degree of immunohistochemical staining was evaluated by the sum of the staining intensity score (0, no, 1: light yellow, 2: yellow, 3: brown yellow) and the staining proportion score (0, < 25%, 1: 25–50%, 2: 51–75%, 3: > 75%). The p16 protein expression was assessed by the final score (0 ~ 6) of immunohistochemical staining.

### Statistical analysis

SPSS 17.0 software was used throughout. The difference of methylation level between CRC tissue and adjacent normal tissue at each region was analyzed by paired samples T-test (*p16* exon1A-T, exon2A-T) and the difference between *p16* exon 1 and exon 2 was analyzed by independent samples T-test (exon1T - exon2T). Heat-map analysis was carried out by HemI 1.0 software (http://hemi.biocuckoo.org/index.php). The association of *p16* exon 1/2 methylation level and p16 protein expression level in 30 CRC tissues were analyzed using Spearman test. The relationship between the methylation status of each region and the clinicopathological features of 30 CRC patients were analyzed using Fisher’s exact test. A *p* value < 0.05 was considered statistically significant.

## Results

### Both exon 1 and exon 2 within *p16* gene body contain a typical CpG island

**Fig. 1:**
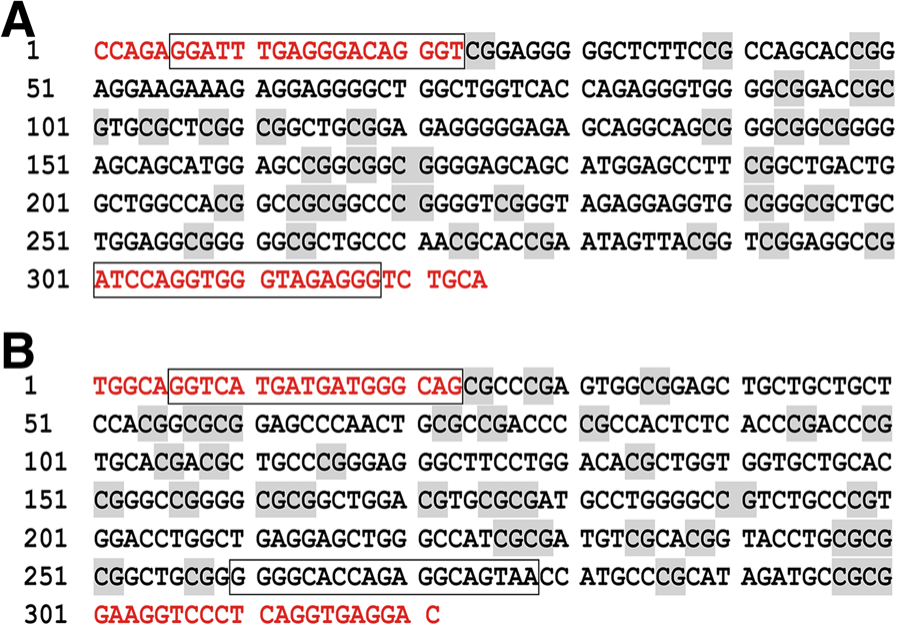
The target CpG-rich regions within *p16* gene body. **a** The CpG island lying in *p16* exon 1 contains 324 nucleotides and 31 CpG sites (grey shading). **b** The CpG island lying in *p16* exon 2 contains 321 nucleotides and 35 CpG sites (grey shading). Using nested PCR with outer primers (the locations were indicated in red type) and inner primers (the locations were indicated by black box), the two target regions were finally amplified, namely one fragment including 313 bp and 31 CpG sites and another including 273 bp and 32 CpG sites

The *p16* gene is located on Chromosome 9: 21,967,753-21,995,301 reverse strand and its transcript variant 1 generates from 3 exons. The sequences of these 3 exons (19,359…19,814, 23,284…23,590, 26,250…26,740) were obtained from the genomic sequence (NCBI Reference Sequence: NC_000009.11) and then analyzed by CpG island searcher tool (http://www.ebi.ac.uk/Tools/seqstats/emboss_cpgplot/). Two typical islands were found in *p16* exon 1 and exon 2 regions. As shown in Fig. 1, one CpG island lying in *p16* exon 1 contains 324 nucleotides and 32 CpG sites, the other lying in *p16* exon 2 contains 321 nucleotides and 35 CpG sites. The two genomic regions (19,509…19,832, 23,278…23,598) covering the whole CpG island were chosen as target regions for evaluating *p16* gene body methylation alterations.

### PCR amplification and DNA sequencing validation

**Fig. 2:**
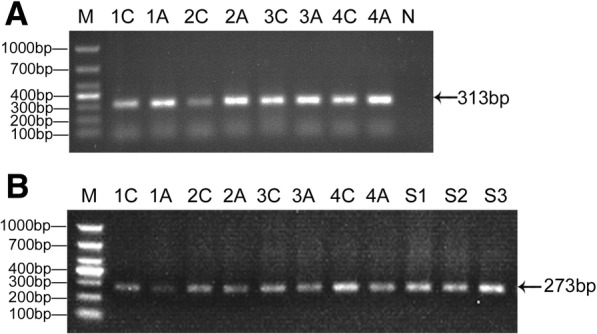
The target CpG islands within *p16* gene body were obtained by nested PCR. **a** The CpG island lying *p16* exon 1 (313 bp) and (**b**) The CpG island lying *p16* exon 2 (273 bp) were amplified from genomic DNAs of paired tissues from CRC patients, respectively. M: DL1000 DNA Marker (Takara, Dalian); 1C~4C: colorectal cancer tissues; 1A~4A: adjacent normal tissues to corresponding carcinoma; N: negative control; S1~S3: three specific DNA samples with known methylation levels as controls of methylation detection

From the gel electrophoresis results shown in Fig. 2, bisulfite PCR amplification of two exon regions and three specific DNA samples (with known methylation levels as controls of methylation level detection) were successfully implemented using nested PCR with modified primers. The sizes of all PCR products were in accordance with expectation.

To further confirm the correctness of the PCR products, Sanger sequencing with inner PCR primers was applied to PCR products from three individual tissues. Sequence alignment revealed that the sequences of above PCR products were accordant with the original sequences of corresponding target regions except for the C-T converted sites, which verified the accuracy of PCR amplification.

### LC-MS/MS analysis of nucleobases

**Fig. 3:**
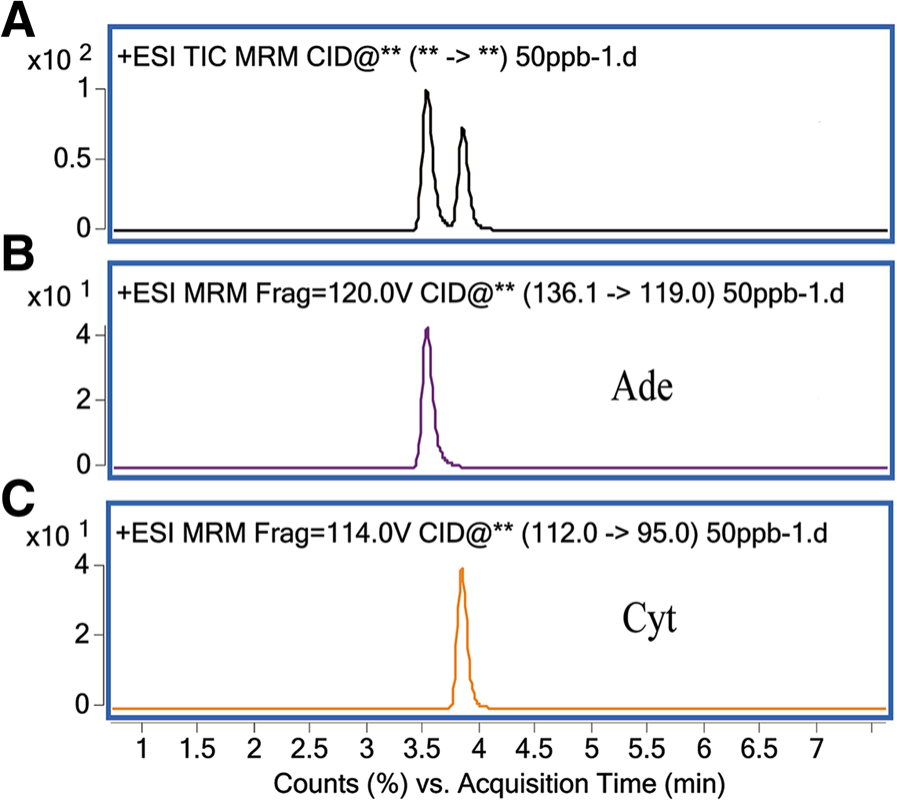
The multiple reaction monitoring chromatograms of Adenine and Cytosine. **a** Total ions chromatograph (TIC). **b** Adenine, m/z 136.1 > 119.0. **c** Cytosine, m/z 112.0 > 95.0

The purified PCR products from different samples were hydrolyzed by formic acid and then analyzed by LC-MS/MS. Nucleobase quantification was accomplished in multiple reactions monitoring (MRM) mode. The mass chromatogram showed that all the analytes exhibited favorable peak shape. Cyt and Ade were completely separated in 4 min (Fig. 3). The methylation level of three control DNA samples were firstly measured using the LC-MS/MS approach to validate experiment condition. The detection results were highly consistent with those identified by the golden standard method bisulfite sequencing PCR (BSP). These results demonstrated that the LC-MS/MS method and experiment condition could be used to evaluate the methylation levels of *p16* exon 1 and exon 2 in the following tissue samples.

### The methylation levels and differences of *p16* exon 1 and exon 2 in CRC

**Fig. 4:**
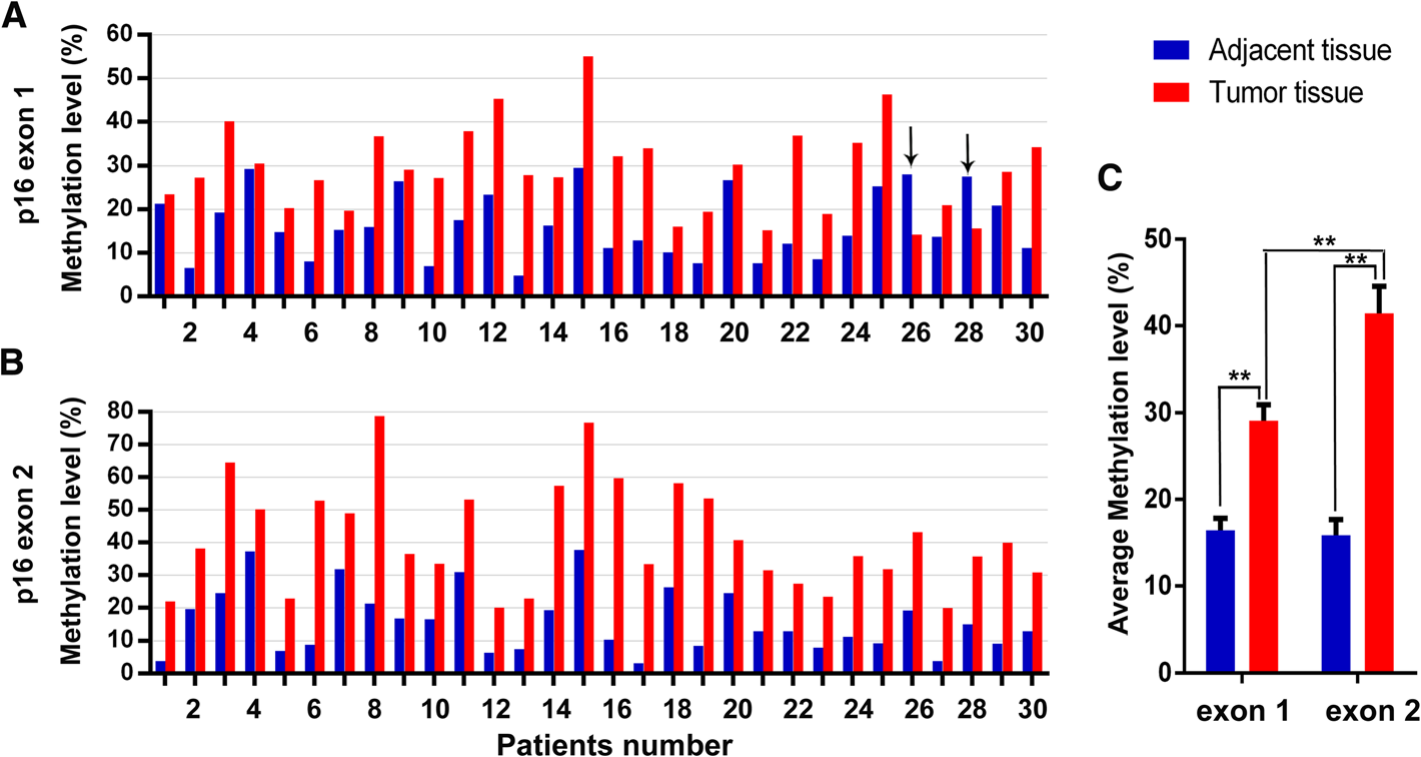
The methylation levels of *p16* gene body in 30 CRC patients. **a** The methylation levels of *p16* exon 1 region. **b** The methylation levels of *p16* exon 2 region. Each sample was measured three times. **c** The methylation differences between *p16* exon 1 and exon 2. Data was means ± standard errors. **p < 0.01

By the established LC-MS/MS method and experiment conditions, tumors and corresponding adjacent normal tissues from 30 CRC patients were evaluated for the methylation level of both *p16* exon 1 and exon 2 regions. The results were shown in Fig. 4a and b. The methylation level of *p16* exon 1 in adjacent normal tissues ranged from 4.77 to 29.53% (mean 16.41%; median 15.28%), while in tumor tissues they varied from 14.19 to 55.07% (mean 29.11%; median 27.91%). Comparing with *p16* exon 1, the methylation level of *p16* exon 2 ranged more widely, from 3.22 to 37.73% in adjacent normal tissues (mean 15.88%; median 12.85%) and from 19.89 to 78.67% in tumor tissues (mean 41.44; median 38.14%).

As a whole, tumors were more highly methylated than adjacent normal tissues at both exon 1 and exon 2 regions with statistical significance as shown in Fig. 4c, namely *p16* exon 1 adjacent versus tumor (*t* = 6.579, *p* < 0.01) and *p16* exon 2 adjacent versus tumor (*t* = 11.543, *p* < 0.01). In tumors, the average methylation level of *p16* exon 2 was significantly higher than *p16* exon 1 (*t* = 3.544, *p* < 0.01). It was noteworthy that there were 2 cases (Patient No. 26 and 28) showed greater methylation in the adjacent tissue than in carcinoma tissue at *p16* exon 1 region, but they were the opposite at *p16* exon 2 region. Paired samples analysis found a significant correlation in *p16* exon 2 methylation between tumors and adjacent tissues (*r* = 0.667, *p* < 0.01), while *p16* exon 1 did not show this correlation.

**Fig. 5:**
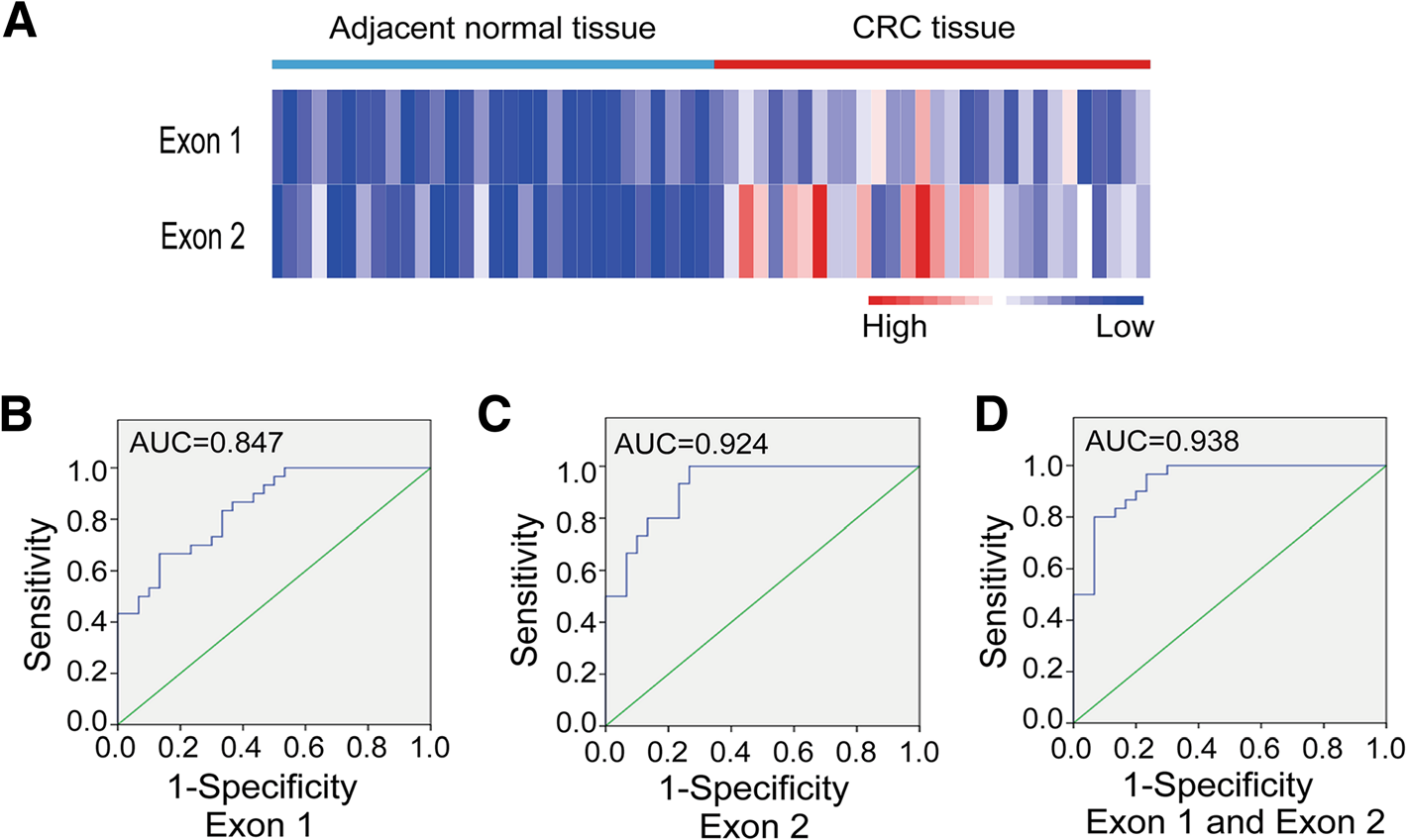
The *p16* gene body methylation as potential biomarker of CRC. **a** Heat-map of the methylation of *p16* exon 1 and exon 2 in CRC tissues and adjacent normal tissues (*p < 0.05). **b** ROC curve analysis of the *p16* exon 1 methylation for distinguishing CRC and adjacent normal tissues. **c** ROC curve of the *p16* exon 2 methylation between two groups. **d** ROC curve analysis of the simultaneous use of *p16* exon 1 and exon 2

Additionally, heat-map and ROC curve analysis revealed that *p16* exon 2 had an excellent sensitivity and specificity, and it was better than exon 1 (Fig. 5) for distinguishing adjacent normal tissue and CRC tissue. The simultaneous use of two indicators could promote the sensitivity and specificity, showing a powerful potential as biomarker for CRC diagnosis.

### Association between *p16* exon 1/2 methylation level and p16 protein expression

**Fig. 6:**
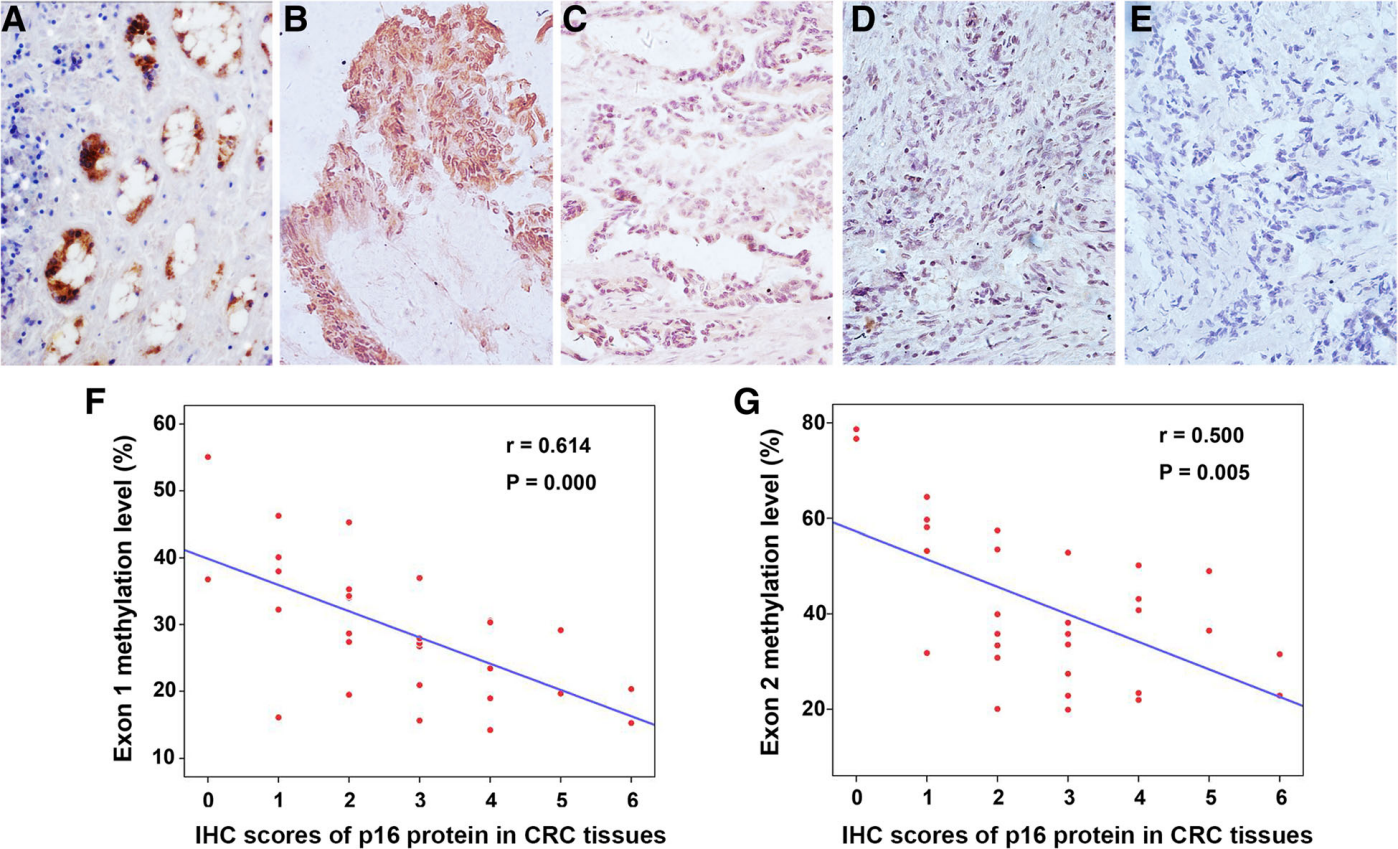
p16 protein expression in CRC tissues by immunohistochemistry (IHC) and its association with *p16* exon 1/2 methylation level. **a** Example of high p16 expression in adjacent normal tissue. **b** Example of high p16 expression in a CRC tissue without aberrant hypermethylation in both exon 1 and exon 2. Example of moderate p16 expression in a CRC tissue with aberrant hypermethylation in either exon 1 (**c**) or exon 2 (**d**). **e** Example of negative p16 expression in a CRC tissue with aberrant hypermethylation in both exon 1 and exon 2. **f/g** The correlation analysis of p16 protein expression and *p16* exon 1/2 methylation level

The expression of p16 protein in CRC tissues was analyzed by immunohistochemistry. As shown in Fig. 6, p16 expression in adjacent normal tissues was higher than that in CRC tissues (Fig. 6a vs Fig. 6b–e). Moreover, p16 protein expression tended to be lower as *p16* methylation level increased in CRC tissues (Fig. 6b^–^e). Statistical analysis revealed that p16 protein expression was negatively related to the methylation level of exon 1 (*r* = 0.614, *p* = 0.000) (Fig. 6f) and exon 2 (*r* = 0.500, *p* = 0.005) (Fig. 6g).

### Association between p16 exon 1/2 hypermethylation and clinicopathological features

Although the average methylation level in non-neoplastic tissues was low at both *p16* exon 1 and exon 2 regions, in some cases it could reach > 30% and far beyond the average. This non-negligible methylation pattern suggests that corresponding normal tissue must be used as a control in the assessment of *p16* hypermethylation in CRC. In this work, using a threshold value of 20% methylation difference between tumor and adjacent normal tissue, all clinical cases were classified into two categories as follows: negative (difference < 20%) and positive (difference ≥ 20%) aberrant hypermethylation group. Accounting for, 14 patients at *p16* exon 1 and 15 patients at *p16* exon 2 were considered positive aberrant hypermethylation, respectively, of 30 CRC cases.

Nextly, the relationship between the methylation status of each region and gender, age and T/N/Dukes stage were analyzed statistically, respectively, to explore the correlation between *p16* exon 1/2 hypermethylation and clinicopathological features. Results were shown in Table 2. A statistically significant association between *p16* exon 1 hypermethylation and N/Dukes stage were found (*p* = 0.033), in which pN1–2 or Dukes C stage showed more frequently hypermethylation than pN0 or Dukes A/B stage. Moreover, a significant correlation between *p16* exon 2 hypermethylation and T stage was also observed (*p* = 0.035). Non-significant association was observed between the methylation of each region with gender, age or differentiation.

**Table 2:** The relationship between CRC clinicopathological features and *p16* exon 1/2 hypermethylation

Variables	N	*p16* exon 1 hypermethylation (n; %)		*P-*value	*p16* exon 2 hypermethylation (n; %)		*P-*value
		Negative	Positive		Negative	Positive	
Gender							
Female	12	6 (50%)	6 (50%)	1.000	8 (66.7%)	4 (33.3%)	0.264
Male	18	10 (55.6%)	8 (45.4%)		7 (38.9%)	11 (61.1%)	
Age							
≤ 55 years	15	6 (40%)	9 (60%)	0.272	6 (40%)	9 (60%)	0.466
> 55 years	15	10 (66.7%)	5 (33.3%)		9 (60%)	6 (40%)	
Differentiation							
Low	6	1 (16.7%)	5 (83.3%)	0.072	1 (16.7%)	5 (83.3%)	0.169
Moderate-High	24	15 (75%)	9 (25%)		14 (58.3%)	10 (41.7%)	
pT stage							
pT1–2	8	6 (75%)	2 (25%)	0.226	7 (87.5%)	1 (12.5%)	0.035*
pT3–4	22	10 (45.5%)	12 (54.5%)		8 (36.4%)	14 (63.6%)	
pN stage							
pN0	13	10 (76.9%)	3 (23.1%)	0.033*	8 (61.5%)	5 (38.5%)	0.462
pN1–2	17	6 (35.3%)	11 (64.7%)		7 (41.2%)	10 (58.8%)	
Dukes stage							
A-B	13	10 (76.9%)	3 (23.1%)	0.033*	8 (61.5%)	5 (38.5%)	0.462
C-D	17	6 (35.3%)	11 (64.7%)		7 (41.2%)	10 (58.8%)	

*Statistically significant

## Discussion

It is largely accepted that *p16* promoter hypermethylation occurs frequently in CRC. However, its clinicopathological significance remains controversial because of the inconsistent research results. In previous studies, the methods commonly used to quantify DNA methylation included methylation specific PCR (MSP) [9, 11, 13, 17, 28] and quantitative MSP [10, 20], MethyLight [12, 19] and methylation-sensitive high resolution melting (MS-HRM) [29], BSP [15] and pyrosequencing [16]. Some of them analyzed one or several CpG sites, and others analyzed a genomic region with some length limitation about < 200 bp. Moreover, different studies targeted different CpG sites or genomic regions. These methodological factors resulted in large differences and non-comparability, which may be one of important reasons for inconsistent results of *p16* PHM in previous researches.

To address this issue, we adopted a LC-MS/MS approach in present study, which was recently reported [30, 32]. Although the LC-MS/MS method can’t distinguish the methylation status of single CpG, it can provide an average methylation level across all CpG sites of a target region and make it easy to compare the methylation alteration between different samples. A significant characteristic of this method is no limitation on fragment length and CpG density/number, which permits to detect a whole CpG island (usually 200 ~ 3000 bp) and to analyze more CpG sites at one time. The detection results of three methylation controls demonstrated it covered a wide detection range (from 0 to 100% methylation) and had a high accuracy.

Therefore, we adopted this LC-MS/MS approach to determine methylation levels of the whole CpG islands within *p16* gene body in CRC tissues. We found that the overall methylation levels of two CpG-rich regions were both significantly higher in tumors than in adjacent normal tissues. Comparing with *p16* exon 1, higher and more frequent hypermethylation occurred at *p16* exon 2 in tumors. It’s worth noting that there were 2 cases showed higher methylation in adjacent normal tissue than carcinoma tissue at *p16* exon 1 region, being in line with previous pyrosequencing result of *p16* promoter-exon1 region [16]. This fact might be an important reason for the controversy of *p16* promoter hypermethylation as CRC biomarker. ROC curve analysis revealed that *p16* exon 2 had a high sensitivity and specificity for distinguishing adjacent normal tissue and CRC tissue, and the combination use of both indicators could further improve the sensitivity and specificity. These results suggested that longer genomic region covering more CpG sites could better reflect the overall methylation status of some specific gene, and therefore could be better indicators.

In our research, aberrant hypermethylation of *p16* exon 1 or exon 2 were observed in only about 50% of the CRCs, which were similar with previous studies [27, 29]. This frequency is relatively modest compared to some other loci such as ADAMTS19 [33], and it may be a limitation as biomarkers. However, combining two loci, it reached 73.3% when either exon 1 or exon 2 was aberrant hyper-methylated. ROC curve also showed the combined use of two loci could promote the sensitivity and specificity. Concerning the importance of *p16* gene in tumorigenesis, our findings supported that the combination of *p16* exon 1 and exon 2 could be an effective methylation marker of CRC.

We further explored the relationship between the aberrant hypermethylation of *p16* gene body and clinicopathological features of CRC patients. Because *p16* methylation may occur in non-neoplastic tissues, a threshold should be set to confirm positive hypermethylation. Our results showed the average methylation differences between tumors and adjacent normal tissues of exon 1 and exon 2 both reached 10% (Fig. 4c). Therefore, we set a threshold value of 20% (two fold of 10%) to ensure that only aberrant hypermethylation cases were assigned as positive. This threshold value was in line with an previous systematic study [16]. Considering cases with at least 20% methylation difference between tumor and normal tissue as positive, all clinical cases were classified into two categories. Subsequent statistical analysis uncovered a significant correlation between *p16* exon 1 and N/Dukes staging, also between *p16* exon 2 and T staging, which suggested the hypermethylation of *p16* gene body was associated with CRC invasion and metastasis. These findings further supported that the combination of *p16* exon 1 and exon 2 could be an effective methylation marker of CRC.

Currently, methylation alteration of exon-based gene body has attracted more attention since GbM was found that frequently occurred in some oncogenic genes and DNA methylation in transcribed regions were also correlated with gene expression [25, 26]. It would provide more detectable loci and may be novel biomarkers or therapeutic targets in cancer [26, 32]. Here, our study observed that the methylation level of *p16* gene body had high sensitivity and specificity as potential CRC biomarker, and the hypermethylation of *p16* exon 1 or exon 2 was associated with N/Dukes or T staging. The immunohistochemistry assay demonstrated a negative correlation between *p16* exon 1/2 methylation level and p16 protein expression. These results suggested that the gene body methylation could affect *p16* gene expression, possibly by preventing aberrant transcription initiation or effecting transcription elongation, and thus be associated with CRC progression. These findings will promote the application of *p16* gene body methylation as biomarker for CRC diagnosis.

## Conclusions

In summary, the hypermethylation of exon 1 and exon 2 within *p16* gene body were confirmed in CRC by a LC-MS/MS strategy. The methylation of *p16* exon 1 and exon 2 had good potentials for distinguishing CRC tumor and adjacent tissue, and the simultaneous use of both indicators could further promote the sensitivity and specificity. The methylation level of *p16* exon 1/2 was negatively related to p16 protein expression. The hypermethylation of *p16* exon 1 was associated with N/Dukes staging, and that of *p16* exon 2 was associated with T staging. The combination of *p16* exon 1 and exon 2 could better reflect the overall methylation status of *p16* gene body and may be a more reliable methylation biomarker of CRC. These results provide a new insight into the *p16* gene body methylation as biomarkers for CRC diagnosis.

## Abbreviations

BSP: bisulfite sequencing PCR
CRC: colorectal cancer
GbM: gene body methylation
LC-MS/MS: liquid chromatography tandem mass spectrometry
MRM: multiple reactions monitoring
PHM: promoter hypermethylation
